# Supplementary material for: Protocol for a saline-free surgical preparation of adult Drosophila for chronic in vivo brain imaging
Source: STAR Protoc. 2026 May 7;7(2):104550. doi: 10.1016/j.xpro.2026.104550 (PMC13186001; doi:10.1016/j.xpro.2026.104550)
Supplement: Document S1. Tethering station assembly guide, related to before you begin [file mmc1.pdf]

Tethering-Station-Assembly

Instructions for building the inexpensive tethering station

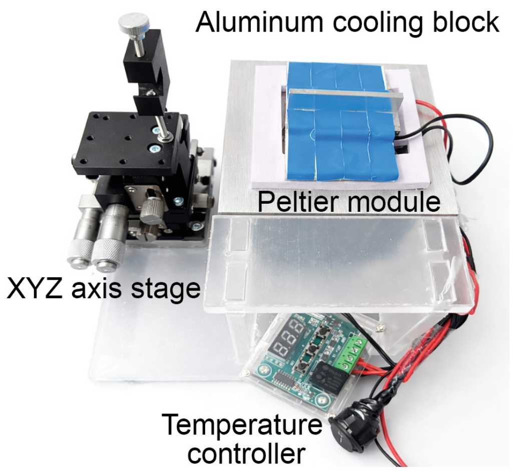

Figure 1: Fly tethering station.

Table of Contents

- 1. Prepare all needed parts
- 2. Assemble the acrylic frame
- 3. Assemble the Peltier cooler
- 4. Mount the micromanipulator
- 5. Install the thread adapter and Mini-V clamp
- 6. Install the fly-holding rod and fly holder
- 7. Power on and set the cooler temperature
- 8. Required parts list

Assembly Steps

1 Prepare all needed parts

1.1 Verify purchasable items

- Confirm you have all **purchasable components** listed in the **Required Parts List (PPL)** table.
- Purchase any missing items before starting the build.

1.2 Prepare custom acrylic parts

- You need to prepare:
  - a. Acrylic fly holder.

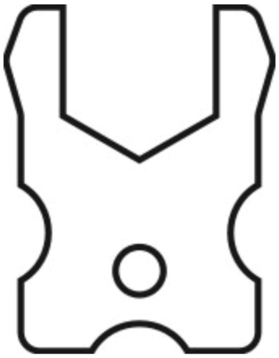

Figure 2: Acrylic fly mount, 1.5mm (1/16”) thickness.

- b. Acrylic assembly for the tethering station.

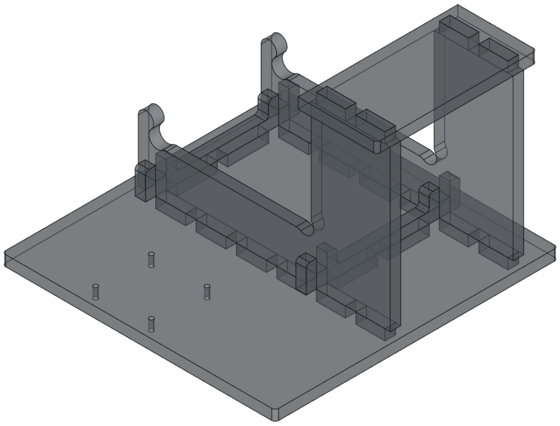

Figure 3: CAD rendering of the tethering station's acrylic assembly, designed in FreeCAD.

- Obtain these acrylic-sheet parts by either:
  - Laser-cutting the acrylic sheets yourself, or
  - Ordering laser-cut parts from a service (e.g., Ponoko).
- Check acrylic thickness before cutting/ordering. The SVG filenames indicate the required thickness:
  - **1.5 mm (1/16”)**
  - **6 mm (1/4”)**
- Cut paths only: all paths in the SVG files are intended to be **cut**, not engraved.
  - Download fly holder SVG files
  - Download tethering station SVG files

1.3 Tap threads in the baseplate

- Tap **M3 threads** into the baseplate holes used to mount the micromanipulator.

1.4 Prepare 3D-printed parts

- Obtain the 3D-printed parts by either:
  - Printing them yourself, or
  - Ordering them from a service (e.g., Craftcloud).
- Recommended filament:
  - **PLA** (works well for most uses)
  - **ASA** (recommended if you need improved UV resistance)
- Suggested print settings:
  - **0.2 mm layer height**
  - **No supports**
- Mini-V clamp: insert **M3 nuts** into both slots.

2 Assemble the acrylic frame

- Assemble the main structure using the acrylic parts, following the render in **Figure 3**.
- Fasten all acrylic joints with **cyanoacrylate adhesive** (super glue), as shown in **Figure 3**.

Optional: add rubber feet

For a better grip between the baseplate and the table, attach **four rubber feet** under the baseplate—**one at each corner**.

3 Assemble the peltier cooler

3.1 W1209 temperature controller

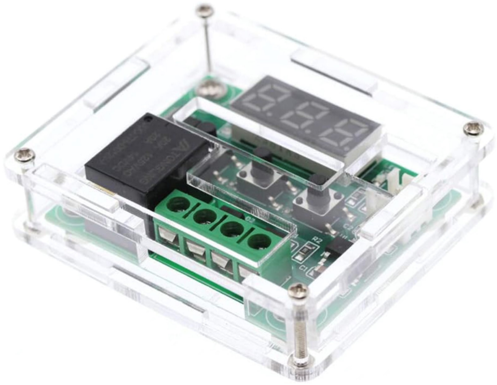

Figure 4: HiLetgo 2pcs W1209 with Case 12V DC Digital Temperature Controller.

- Assemble the **W1209 temperature controller (PPL)**.
- If your unit includes an acrylic housing, assemble the housing first.

> **Wiring safety note**
>
> Do **not** splice wires by twisting metal-on-metal only. Poor splices can overheat under load. Use one of the following:
>
> - solder + heat-shrink, or proper connectors (e.g., crimp connectors or
> - WAGO lever nuts).

3.2 Wire the temperature controller to the peltier cooler

Prepare:

- one **3” red wire** (cut + strip ends) one **10”**
- **black wire** (cut + strip ends)
- **12 V power supply female**
- **barrel connector rocker**
- **switch Peltier cooloer**
-

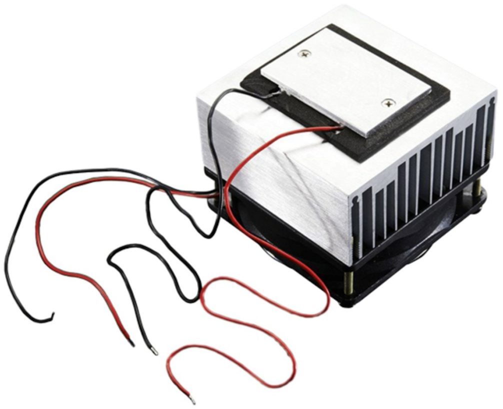

Figure 5: Thermoelectric, Peltier Assemblies.

Wire as follows: a. Use the **3” red wire** to connect terminals **K1 → 12V** on the W1209. b. Connect one end of the **10” black wire** to **GND** on the W1209. c. Connect the other end of the **10” black wire** to the **negative (−)** terminal of the **female barrel connector**. d. Connect the **black wire** of the **rocker switch** to **12V** on the W1209. e. Connect the **red wire** of the **rocker switch** to the **positive (+)** terminal of the **female barrel connector**.

Cooler load wiring (Peltier + fan assembly):

- **Red wires → K0 Black wires → GND**
- You can wire the cooler load either **now** or **after** mounting the cooler to the acrylic structure.

3.3 Prepare and mount the aluminum T-bar

a. Cut a **40-mm-length** piece of the **aluminum T-bar** (a bandsaw works well). b. Apply **thermal paste** to the bottom face of the T-bar (orientation as in **Figure 1**). c. Place the **NTC temperature probe** so it touches **both**:

- the rim of the T-bar, and
- the cold plate
  Add thermal paste to thermally couple the probe to both surfaces. d. Secure the T-bar using **thermal tape** (use **4 × 10 mm** strips) as shown in **Figure 6**.

- You do **not** need to remove the blue liner from the tape after applying it.

- 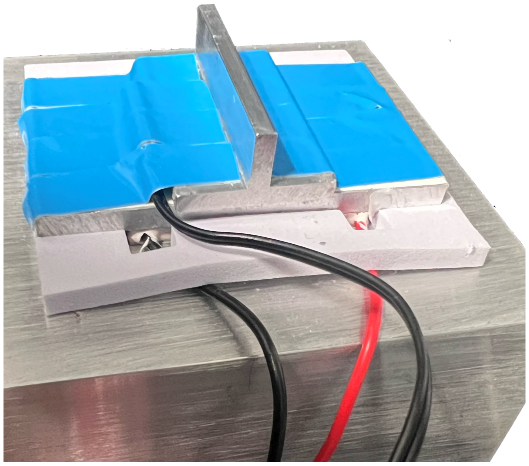

Figure 6: The aluminum T-bar on the cooler

3.4 Seat the cooler on the acrylic frame

- Place the cooler into its seat as shown in **Figure 1**.
- Optionally use **cyanoacrylate adhesive** (super glue) for additional fastening.

4 Mount the micromanipulator

a. Place the micromanipulator on the acrylic baseplate **with the 6 mm raising platform underneath**. b. To access the screw mounts:

- Slide the lowest platform to an **edge position** to expose the screw holes on the opposite side. c. Insert **M3 × 12** screws and fasten the micromanipulator into the baseplate. d. Move the platform to the other edge position and repeat to install the remaining screws.

5 Install the thread adapter and Mini-V clamp

a. Screw your chosen **thread adapter** into the top surface of the micromanipulator. b. Wrap **one layer** of **PTFE (thread seal) tape** or **electrical tape** around the exposed threads to add friction (prevents the clamp from rotating freely). c. Attach the **Mini-V clamp** above the thread adapter.

Notes by clamp version 3D-printed version (shown in Figure 1)

- The “thread adapter” is an **M3 × 36** threaded rod.
  - We made this by cutting an **M3 × 40** screw down to **36 mm** using a rotary tool + cutoff wheel (other methods are fine).
- Fasten the rod by: a. threading it into the micromanipulator platform hole closest to the T-bar, then b. adding a **washer + nut** to lock it in place.

Thorlabs-parts version

- You may need **M3 standoffs** to reach the height of the aluminum T-bar apex. This was not
- tested; an M3 standoff kit is included in the PPL as an option.

6 Install the fly-holding rod and fly holder

a. Insert the appropriate **thumb screw** into the top of the Mini-V clamp (type depends on your clamp choice). b. Insert the **fly-holding rod** (Thorlabs or 3D-printed) into the Mini-V clamp and secure it with the thumb screw. c. Attach the acrylic fly holder to the rod using one of the following:

- **M2.5 screw + nut**, or
- **#2-56 screw + nut** d. Use the small hole at one end of the fly-holding rod for the fly holder fastener.

7 Power on and set the cooler temperature

a. Plug the **12 V power supply** into the **female barrel connector**. b. Turn on the **rocker switch** when you are ready to cool.

Recommended temperature setpoint

- Target: keep the **cold plate ~0 °C**.
- The NTC probe may not report the cold-plate temperature accurately.
- Using an IR camera, we found that setting the controller to **+10 °C** produced the desired cold-plate temperature (~0 °C).

How to set the temperature on the W1209

a. **Click** (do not long-press) the **SET** button, which will make the display flash. b. Use **+** and **–** to adjust the set temperature. c. Click the SET button again to set the displayed temperature.

8 Required parts list

Name	Quantity	Description	Notes	Link
Tap Wrench Tool Set	1	Mesee 6 Pcs Tap Wrench Tool Set, Adjustable T-Handle Tap Holder Wrench with Metric Plug Taps (M3–M8)		Amazon
Super Glue	1	Loctite Super Glue Professional Liquid, 20 g		Amazon
Temperature Controller	1	HiLetgo W1209 (12V) Digital Temperature Controller (with case)		Amazon
WAGO Lever Splice	5	Push-in butt splice connector, 12–24 AWG, transparent/orange		DigiKey
Black Wire	1	22 AWG hook-up wire, black, 25 ft (7.62 m)		DigiKey
**Name**	**Quantity**	**Description**	**Notes**	**Link**
Red Wire	1	22 AWG hook-up wire, red, 25 ft (7.62 m)		DigiKey
Rocker Switch	1	Rocker switch, SPST, 20A (AC) 125V, panel mount, snap-in		DigiKey
Thermal Tape	1	Double-sided thermal adhesive tape, 25 m × 10 mm × 0.20 mm		Amazon
Thermal Paste	1	Thermal silicone compound, 1 g syringe		DigiKey
Cooler	1	Direct-to-air thermoelectric assembly		DigiKey
Power Supply	1	12V 8A (96W) AC/DC power adapter		Amazon
Micro-Manipulator	1	XYZ axis manual precision linear stage (40 × 40 mm)		Amazon

Bumper Feet	4	Square tapered bumper feet (polyurethane)	Optional; example part	DigiKey
Aluminum T-Bar	1	6061 aluminum T-bar, 1/2” high × 1” wide, 1/8” wall	Buy 2 ft; cut a 40 mm section (bandsaw)	McMaster
Plate-Holding Rod (Thorlabs option)	1	ER90C - 90° “T” Extension	Or 3D print	Thorlabs
Cage Rod (for plate-holding rod)	1	ER2 - Cage Assembly Rod, 2” long, Ø6 mm	Used with ER90C	Thorlabs
Electrical Tape (McMaster)	1	Electrical tape, 3/4” wide, 60 ft, black		McMaster
Electrical Tape (DigiKey)	1	Electrical tape, PSA adhesive, black, 3/4” × 60’	Alternative source	DigiKey
PTFE Seal Tape	1	PTFE thread seal tape (plumber’s tape), 1/2” × 520”		Amazon

Clamp Options

Option A: Imperial (8-32)

Name	Quantity	Description	Notes	Link
Thumb Screw	1	#8-32 hex head thumb screw, hex drive, nylon		DigiKey
Thread Adapter	1	Male hex thread adapter, 8-32 to M3 × 0.5		McMaster
Clamp	1	VH1 - Miniature V-Clamp, 0.42” long, 8-32 tapped		Thorlabs
M3 Standoff Kit	1	M3 standoff assortment kit	Optional height tuning	Amazon

Option B: Metric (M4)

Name	Quantity	Description	Notes	Link
Thumb Screw	1	#8-32 hex head thumb screw, hex drive, nylon		DigiKey
Thread Adapter	1	Male hex thread adapter, M3 × 0.5 to M4 × 0.7		McMaster
Clamp	1	VH1/M - Miniature V-Clamp, 0.42” long, M4 tapped		Thorlabs
M3 Standoff Kit	1	M3 standoff assortment kit	Optional height tuning	Amazon

Option C: Metric (3D-printed clamp)

Name	Quantity	Description	Notes	Link

Thumb Screw	1	Knurled thumb screws, M3 × 20		Amazon
Thread Adapter	1	M3 × 36 threaded rod	Fabricate per procedure (e.g., cut down an M3 × 40)	

Fasteners

Name	Quantity	Description	Notes	Link
Screws (M3 × 12 mm)	1	316 stainless button head hex drive screws, M3 × 0.5, 12 mm		McMaster
Nuts (M3)	1	Zinc-plated steel hex nut, M3 × 0.5		McMaster
Washers (M3)	1	18-8 stainless washer for M3 (3.2 mm ID, 7 mm OD)		McMaster
M3 Fastener Kit	1	M3 screw/nut/washer assortment kit	Alternative to individual parts	Amazon
Screw (#2-56)	1	#2-56 pan head slotted drive nylon screw	For acrylic fly holder fastening	DigiKey
Nut (#2-56)	1	#2-56 hex nut, nylon	For acrylic fly holder fastening	DigiKey

Acrylic Sheets (if cutting in-house with a CO_2 laser)

Name	Quantity	Description	Notes	Link
Acrylic sheet (1/16", 1.5 mm)	1	Clear scratch- and UV-resistant acrylic sheet, 12" × 12" × 1/16"	For 1.5 mm SVG parts	McMaster
Acrylic sheet (1/4", 6 mm)	1	Clear scratch- and UV-resistant acrylic sheet, 12" × 12" × 1/4"	For 6 mm SVG parts	McMaster
